# Cardiac surgery under cardiopulmonary bypass in pregnancy: report of four cases

**DOI:** 10.1186/s13019-021-01650-4

**Published:** 2021-09-25

**Authors:** Youhao You, Shenghua Liu, Zhaohong Wu, Dunjin Chen, Gefei Wang, Gangdong Chen, Youguang Pan, Xing Zheng

**Affiliations:** 1 Thoracic and Cardiovascular Surgery, The Third Affiliated Hospital of Guangzhou Medical University, Guangzhou, Guangdong China; 2 Obstetrics and Gynecology Department, The Third Affiliated Hospital of Guangzhou Medical University, Guangzhou, Guangdong China

**Keywords:** Heart valve diseases, Cardiac surgical procedure, Cardiopulmonary bypass, Pregnancy, Multidisciplinary team, Perioperative management

## Abstract

**Background:**

Open heart surgery during pregnancy is relatively rare at home and abroad, and there is a higher risk and probability of maternal and infant death. How to carry out heart valve replacement under cardiopulmonary bypass (CPB) under the premise of ensuring the safety of mother and child is the focus of attention at home and abroad.

**Case introduction:**

We reported four cases of cardiac surgeries under CPB during pregnancy performed in our hospital from March 2020 to March 2021. Two of the patients continued their pregnancy after cardiac surgery under CPB. Three patients had infective endocarditis and the other one had an ascending aortic aneurysm. Three patients underwent heart valve placement with the mechanical mitral valve when the other one underwent Bentall surgery. The operations of four cases were all successful, and further follow-up evaluation of the pregnant women and fetuses showed no abnormalities. The patients' detailed information is shown in the following table.

**Conclusion:**

Heart disease during pregnancy should be treated actively and proactively when the patient has obvious symptoms. Heart valve replacement under CPB will be the first choice, and this may become the primary surgical treatment for symptomatic heart disease during pregnancy.

## Background

The incidence of heart disease in pregnant women is 1–4% approximately [1]. Heart disease complicates more than 1% of pregnancies and accounts for 15% of maternal mortality, the leading cause of indirect obstetric deaths [2, 3]. Even in developed countries, the maternal mortality rate has not declined either [2]. Pregnancy is an important factor that aggravates undetected heart disease and makes pregnant women show obvious symptoms. Due to the lack of medical and knowledge and awareness, heart disease in pregnant women is often not detected until the hemodynamic decompensation stage. At this point, cardiac surgical intervention is inevitable [4]. In principle, the cardiac surgical procedures should be avoided during pregnancy, preferably at 6 weeks after delivery. However, when these pregnant women develop obvious symptoms, early intervention of cardiac surgical procedures during pregnancy is necessary. Setting up a multidisciplinary team (MDT) for management is a crucial step, which is also consistently recommended in the guidelines developed by the European Society of Cardiology (ESC). As a countrywide intensive maternal care center in China, our hospital often has such special cases of heart disease during pregnancy, but only a small number of pregnant women undergo cardiac surgery under CPB during pregnancy. To provide more clinical treatment experience, including preoperative, intraoperative, and postoperative points of concern, we reported these four cases, which successfully underwent cardiac surgery under CPB during pregnancy.

## Case report

### Patient's general information

The four patients were aged between 24 and 34 years old, with a gestational age of 23–34 weeks. And the average hospitalization time for these four patients was 34 days. Three patients had IE with vegetation formation and severe mitral valve regurgitation, which all had bacteria cultured from blood. The patient's characteristics are listed in Table 1.

**Table 1:** Patient's characteristics

Patient no	Age (y)	Weeks of gestation during surgery (w)	Type of heart disease	Date of admission	Date of discharge	Time of hospitalization (d)	The type of bacteria (blood culture)
1	34	27	IE (Vegetation formation, mitral valve prolapse, perforation, and SMVR)	2020–03-31	2020–05-15	45	Oral streptococcus, Staphylococcus Haemolyticus
2	24	23	IE (Vegetation formation, mitral valve prolapse, perforation, and SMVR)	2020–06-20	2020–07-21	31	Streptococcus Parasanguis
3	29	33	Aneurysm of ascending aorta (about 10 cm in diameter), SAVR	2020–05-14	2020–05-29	15	–
4	28	34	IE (Vegetation formation, mitral valve prolapse, perforation, and SMVR)	2021–01-26	2021–03-12	45	Hemostreptococcus

y, Year; w, week; d, day; IE, infective endocarditis; SMVR, severe mitral valve regurgitation; SAVR, severe aortic valve regurgitation

### Cardiac surgical procedure, intraoperatory parameters and postoperative treatment management

All these four patients underwent cardiac surgical procedure under CPB, including three mitral valve replacement (MVR), one Bentall procedure. Two patients were performed simultaneous cardiac surgery and cesarean section, and the other two continued their pregnancies after cardiac surgery procedure. According to the particularity of the patients-pregnancy, close intraoperative monitoring was performed, such as aortic cross-clamp time, CPB time, CPB minimum temperature, CPB maximum flow and ACT value at the beginning of CPB, as shown in Table 2. Before the beginning of CPB, we heparinized the patients to achieve an ACT above 480 s and maintained the ACT above 480 s with ACT monitoring. Of course, attention should also be paid to the treatment and management after the procedure, for example, the treatment and monitoring to fetus protection of the two patients who continued to have pregnancy after cardiac surgical procedure, the selection of anti-infective drugs, the course of anti-infective treatment and so on, which are presented in Table 3. Mode of delivery and neonatal outcomes are in Table 4.

**Table 2:** Cardiac surgical procedure and intraoperatory parameters

Patient no	Therapeutic schedule	Cardiac surgical procedure	Aortic cross-clamp time (min)	CPB time (min)	CPB maximum perfusion (ML/min)	ACT value at the beginning of CPB (s)	CPB minimum temperature (℃)	Average temperature during CPB (℃)
1	Cardiac surgery and cesarean section was performed in stages	MVR + TVP	74	126	3.07	541	34.1	35.2
2	Cardiac surgery and cesarean section was performed in stages	MVR + TVP	87	135	2.91	504	34.8	35.6
3	Cardiac surgery was performed at the same time as the cesarean section	Bentall procedure	72	113	-	695	31.5	34.0
4	Cardiac surgery was performed at the same time as the cesarean section	MVR + TVP	76	125	2.80	481	34.1	35.6

CPB, cardiopulmonary by pass; ml, mini liter; min, minutes; MVR, mitral valve replacement; TVP, tricuspid valvuloplasty; ACT, activated clotting time; s, seconds

**Table 3:** Postoperative treatment management

Patient no	Fetus protection treatment	Postoperative fetal monitoring	Duration of fetal monitoring	Anti-infective drugs	Duration of anti-infective treatment	Maternal and fetal outcomes
1	Atosiban, magnesium sulfate	FHR was monitored by Doppler ultrasound, closely prenatal examination	Until the time of labor	Vancomycin, imipenem	6 weeks after surgery	Alive, preterm birth
2	Atosiban, magnesium sulfate	FHR was monitored by Doppler ultrasound, closely prenatal examination	Until the time of labor	Vancomycin, meropenem	6 weeks after surgery	Alive, term birth
3	–	–	–	–	–	Alive, preterm birth
4	–	–	–	vancomycin, meropenem	6 weeks after surgery	Alive, preterm birth

FHR, Fetal heart rate

**Table 4:** Mode of delivery and neonatal outcomes

Patient no	Mode of delivery	Neonatal outcomes
1	Vaginal delivery	The baby was delivered at 36 weeks gestation and weighed 2370 g
2	Cesarean section	The baby was successfully delivered at 40 weeks gestation, weighing 2350 g
3	Cesarean section	The fetus weighed 1860 g
4	Cesarean section	The fetus weighed 2020 g

g, Gram

## Discussion

IE is a fatal disease, which is relatively rare in clinical, but with a high mortality and disability rate [5]. Especially in pregnant women, IE has a high incidence of adverse maternal outcomes, as well as infant loss. Treatment and management of pregnancy-related infective endocarditis should be individualized and multidisciplinary. For example, whether open heart surgery is needed, staging or concurrent surgery, the effect of CPB on the fetus in staging surgery, the operation sequence of concurrent surgery, and intraoperative monitoring points. Additionally, the type of heart disease, severity, cardiac function, gestational age, the wishes of the patients and their family should also be considered [6]. Currently, the experience of clinicians in the treatment of pregnancy-related IE is still insufficient. Here, we reported four cases with heart disease during pregnancy who both underwent cardiac surgical procedures under CPB. Two of the patients underwent simultaneous cardiac surgery and cesarean section, that is cardiac surgical procedures 2 or 2.5 hours after cesarean section, and another two patients continued their pregnancies after cardiac surgery under CPB. In the four cases, the minimum gestational age was 23 weeks and the maximum was less than 37 weeks. Three patients with IE underwent MVR, and one patient with ascending aortic aneurysm underwent Bentall surgery. All these four cases were mechanical valves. Cases 1 and 2 underwent staging heart surgical procedures for fetal protection due to their small gestational age. Progesterone was used to suppress uterine contractions after surgery, while magnesium sulfate and atosiban were used for fetal protection. Fetal heart rate (FHR) was closely monitored during cardiac surgery and continued until the time of labor to ensure fetal stability and to avoid premature delivery. And the department of obstetrics was also involved in the management of postoperative monitoring and treatment of the fetuses. Through follow-up, we learned that the pregnant woman in Case 1 delivered a baby girl naturally at 36 weeks gestation, while another pregnant woman in Case 2 delivered a baby boy by cesarean section at 40 weeks gestation. Staged surgery requires more and more complex monitoring and evaluation, including preoperative, intraoperative, and postoperative. For example, the indications and timing of surgery should be evaluated according to the patient's condition; FHR should be monitored continuously, the duration of CPB should be reduced, and the perfusion flow and pressure of CPB should be improved during the operation; anti-infective therapy should be continued until 6 weeks after operation. For these two patients who continued pregnancy after cardiac surgery, they needed a higher cardiac output to maintain a higher cardiac index (CI). After surgery, we gave them a component blood transfusion, albumin supplementation, nutritional enhancement, and vasoactive drugs to run a higher CI. Therefore, open heart surgery in pregnant women is usually avoided and ideally delayed until 6 weeks postpartum [7]. However, when pregnant women develop obvious symptoms, early cardiac surgical intervention is the best option, and heart valve replacement is the first choice. In the four cases we reported, three of the patients developed life-threatening symptoms and the other one was a high-risk ascending aortic aneurysm. Thus, we formed the MDT, including departments of obstetrics, cardiac surgery, anesthesiology, intensive care, neonatology, and medical services, to discuss the treatment options that would be most beneficial to patients. CPB has a great influence on the fetus, the main influencing factors include the time of CPB heparinization, perfusion fluid temperature, perfusion flow and pressure, as well as maternal temperature. In the case that open heart operation is inevitable for pregnant women, anesthesia management, CPB management, intraoperative fetal monitoring and perioperative management are particularly important [8]. In the two cases reported in our report, with the cooperation of the surgeon, anesthesiologist and nurses, the operation time was only about 4 hours, which greatly reduced the time of CPB and kept the perfusion fluid at room temperature of 35℃ to the greatest extent to reduce the adverse outcome of low temperature on the fetuses. The perfusion was performed with high flow, high pressure and high hematocrit, and the FHR was monitored by Doppler ultrasound throughout the operation. Fetal death most often occurs during the cooling and rewarming phases of CPB [8]. Thus, the risk of fetal death can be greatly reduced by performing the operation at room temperature and controlling the temperature changes during the diversion. In these two patients, the temperature variation during CPB cooling and rewarming was 34.1–36.2 ℃ (the average temperature was 35.2 ℃) in case 1 and 34.8–36.4 ℃ (the average temperature was 35.6 ℃) in case 2. Low molecular weight heparin was used in the early stage of the operation, and warfarin was used until about one week before parturition when the condition of the pregnant women and the fetuses were stable, so that the prothrombin international ratio was maintained at 2.0–3.0. Imipenem combined with vancomycin was also used for anti-infective therapy until 6 weeks after surgery. Of course, the fetuses need to be continued to be closely monitored postoperatively. Case 3 was a patient at 33 weeks of pregnancy, diagnosed with ascending aortic aneurysm complicated with severe aortic valve insufficiency. CTA examination indicated that the dilated diameter of the ascending aorta was more than 8cm, and her immediate family members, her aunts and uncles, had a history of Marfan syndrome. She is at high risk during pregnancy, and early cardiac surgery intervention is needed. Cases 3 and 4 both underwent cesarean section and heart valve replacement at the same time, and both mother and fetuses survived. CPB has a greater impact on the fetus and the fetal mortality rate is high, ranging from 16 to 33% [8]. Therefore, the study of Chandni Patel et al recommended that cesarean section be performed prior to CPB-MVR to improve fetal outcome [8]. Simultaneous cardiac surgery and cesarean section, like cases 3 and 4 in our report, should also consider the increased risk of postpartum bleeding as an important risk factor, which may cause pregnant women to lose their uterus. In the two cases we reported, we used uterine balloon tamponade and bilateral uterine artery ascending branch ligation, combined with intravenous drops of oxytocin to prevent massive postpartum hemorrhage. Replacement of heart valves 2 or 2.5 hours after a cesarean section is also a key step in reducing postpartum bleeding in pregnant women. Reducing the aortic cross-clamp time and CPB time also do help to reduce the risk of cardiac surgical procedures and complications in pregnant women. In these four cases we reported, anti-coagulation on CPB was also using activated clotting time (ACT) monitoring. ACT is considered the gold standard in monitoring anti-coagulation for CPB [9]. Bull and colleagues’ study showed no development of clots in the oxygenator or circuit when ACT was maintained above 300 s [9]. In our study, we maintained ACT above 480 s. After systemic heparinization, CPB was started after ACT reached to 480 s. For the selection of drugs commonly used in cardiac surgery, we routinely followed the guidelines and there were no particular drugs that we had to avoid.

## Conclusion

We have successfully performed cardiac surgery under CPB for four pregnant women with heart disease during pregnancy. Two of them were performed simultaneous cardiac surgery and cesarean section, and the other two continued their pregnancies after cardiac surgery. All four patients survived, both mothers and fetuses. These four cases we reported show that surgical interventions should be carried out actively and proactively for heart disease during pregnancy with obvious symptoms. Of course, we should be careful about the occurrence of postoperative complications. Collaborative management of multidisciplinary teams is a good way.

## Data Availability

The data were presented in the main manuscript.

## Abbreviations

CPB: Cardiopulmonary bypass
IE: Infective endocarditis
MDT: Multidisciplinary team
SMVR: Severe mitral valve regurgitation
MVR: Mitral valve replacement
SAVR: Severe aortic valve regurgitation
TVP: Tricuspid valvuloplasty
ACT: Activated clotting time
FHR: Fetal heart rate
CT: Cardiac index
ml: Mini litre
min: Minutes
g: Gram
y: Year
w: Week
d: Day
s: Second
